# Static mandibular condyle positions studied by MRI and condylar position indicator

**DOI:** 10.1038/s41598-022-22745-5

**Published:** 2022-10-25

**Authors:** Josef Freudenthaler, Stefan Lettner, André Gahleitner, Erwin Jonke, Aleš Čelar

**Affiliations:** Medical University of Vienna, University Clinic of Dentistry, Sensengasse 2a, 1090 Wien, Austria

**Keywords:** Physiology, Anatomy, Medical research

## Abstract

We compared mandibular condyle positions as determined by magnetic resonance imaging (MRI) and a mechanical device, the condylar position indicator (CPI). Both methods assessed 3 mandibular positions in 10 asymptomatic males and 10 asymptomatic females, aged 23 to 37 years, free from temporomandibular disorders: maximum intercuspation, bimanually manipulated centric relation, and the unguided neuromuscular position. Bite registrations were obtained for bimanual operator guidance and neuromuscular position. 3 T MRI scans of both temporomandibular joints produced 3D data of the most superior condylar points in all 3 mandibular positions. Using mounted plaster casts and the same bite registrations, an electronic CPI displayed 3D data of its condylar spheres in these positions. The results showed interclass correlation coefficients ranging from 0.03 to 0.66 (95% confidence intervals from 0 to 0.8) and significantly different condyle positions between both methods (p = 0.0012, p < 0.001). The implications of the study emphasize that condyle position is unpredictable and variable. Its exact knowledge requires radiological imaging and should not rely on CPI assessments.

## Introduction

**Figure 1:**
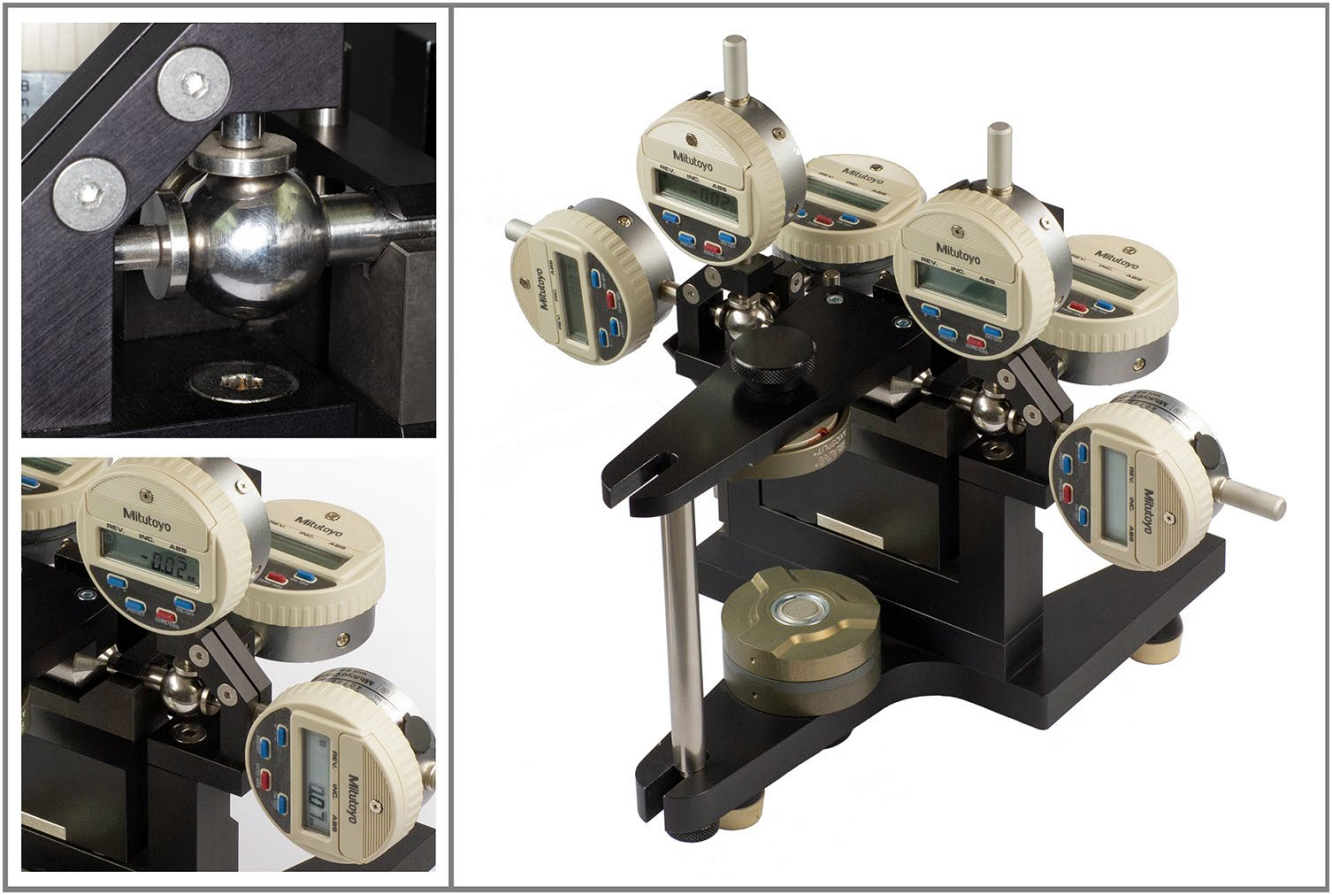
Condylar position indicator without dental casts. Overview and details of condylar sphere and 3D electronic measuring gauges.

The condylar position indicator (CPI) is a modified articulator for instrumental functional analysis of the masticatory organ (Fig. 1)^1^. CPIs quantify differences between two mandibular positions at the condylar levels and the incisal pin. Using bite registrations and mounted casts of the dentition, CPIs measure the slide of the mandible between centric relation and maximum intercuspation or forced bite positions or traumatic occlusal contacts^2,3^. CPIs are also used by maxillofacial surgeons, *e.g.* for determination of postoperative condylar displacement^4^.

As CPIs reproduce condylar positions mechanically, a comparison of the in vitro CPI data with those found in vivo is justified^5^. Magnetic resonance imaging (MRI) epitomizes the state of art for visualisation of articular tissues in vivo^6^ and offers a trustworthy reference for comparison of anatomic structures with mechanical replicas. Consequently, the aim of the current study was to evaluate to which extent MRI and CPI agree in determining condyle positions. Our null hypothesis stated that no differences exist between CPI and MRI condylar position data.

## Subjects, materials and methods

This study was approved by the university ethics commission (Ethikkommission der Medizinischen Universität Wien, ECS1438/2015). The authors confirm that all methods were performed in accordance with the relevant guidelines and regulations. In compliance with the STROBE guidelines on reporting observational studies, recruitment of healthy dental students commenced in the preclinical curriculum over a period of 18 months. All volunteer participants were unaffected by any anamnestic sign and symptom of temporomandibular dysfunction. Seven individuals were excluded from participation because of disc displacement and/or masticatory muscle pain, another 2 could not partake in the MRI scans due to intrauterine metal coils. The definitive sample consisted of 10 women and 10 men. This sample size is in accordance with Kandasamy et al.^7^, whose power analysis yielded n = 19 for 3 different mandibular positions to produce 80% power for detection of mean differences ≤ 1 mm at a significance level of 0.05. The female ages averaged 26.7 ± 2.4y, the male ages 27 ± 3.3y (total range 23–37 years). Informed consent was given after thorough briefing of all planned procedures.

Inclusion criteria embraced full permanent dentition irrespective of 3rd molars, distinct occlusion with unambiguous intercuspation, asymptomatic temporomandibular joints (TMJs) without noise, mandibular movements without limitation (mouth opening > 40 mm, protrusion and laterotrusions ≥ 8 mm), and clear intercuspation of dental casts.

Exclusion criteria encompassed facial trauma, facial pain, TMJ noise, crepitation or disc displacement, mandibular side shift, inflammatory joint disease, TMJ ankylosis, systemic muscle and/or neurological disease, connective tissue disease, orthodontic therapy within the last five years, missing teeth, ambiguous intercuspal position (ICP), systemic medication, pregnancy, use of metal intrauterine contraceptives, claustrophobia, and pathological findings in the MRIs of the now studied TMJs.

Subjective signs and symptoms of facial pain and craniomandibular disorder were evaluated by questionnaire and interview. Further, a single, proficient and experienced clinician palpated head and neck muscles and made multidirectional tractions of the mandible for exclusion of capsulitis and disc displacement. He also tested muscle fatigue or pain during 20-s isometric closure, protrusion, and lateral movements of the mandible. Once the asymptomatic condition was confirmed clinically, the bite registrations were taken during the same appointment. Our protocol stipulated guided and unguided techniques for accomplishing two condylar positions, both not dictated by dental occlusion: bimanual manipulation (BM) and neuromuscular method (NM)^8–12^.

### Bimanual manipulation

A fused double-layered wax plate (1.5 mm Beauty Pink X Hard, Miltex Inc., York, PA, USA) covered the buccal cusps and incisal edges of the upper dental arch without interference with soft tissues. A third wax layer augmented the plate’s anterior part. Examinees sat in a slightly reclined position with their head supported by a headrest, gently holding a cotton roll between upper and lower bicuspids for 5-min deprogramming of the musculature. The examiner removed the cotton roll and positioned the softened wax plate on the air-dried upper teeth. Jaw openings and closures without tooth contact were reiterated 5 times, then the examiner took over and freely hinged the mandible. With his fingers at the posterior and inferior mandibular margins but the thumbs at the chin, the operator pulled the posterior mandible heavily upward and simultaneously pressed the chin downward. The manipulation produced 0.5 to 1 mm impressions in the wax at a vertical bite separation of approximately 2 mm between the first molars. Repetition of the jaw manipulation verified the BM position. Its unambiguous repeatability was given in all 20 examinees.

### Neuromuscular bite registration

NM is a patient-generated technique, which records an unguided mandibular position right before intercuspal contact. For elimination of sensory occlusal contact inputs, 5-min deprogramming of the musculature was accomplished with a cotton roll between upper and lower bicuspids. Patients sat slightly reclined, leaning their head against the dental chair’s headrest. After removal of the cotton, they started slow mandibular up-and-down movements of approximately 3 mm without any antagonist tooth contact during hinging the jaw. The NM position was recorded after 3 min of slow cyclic movements using polyvinyl siloxane (Blue Bite SC, Pluradent, Offenbach, Germany) at 2 mm vertical separation of the first molars. We verified the NM position by immediate repetition, which had to show a clear fit to the first polyvinyl siloxane impressions.

### CPI

Alginate impressions of the dental arches (Orthoprint, Zhermack, Marl, Germany) were poured with plaster (Die-Keen Green, Kulzer, South Bend, IN, USA). After trimming and removal of surface plaster bubbles, we meticulously examined the unambiguous fit of the casts in maximum intercuspation. The SAM anatomical facebow oriented the maxillary casts into a SAM 2P articulator (SAM Präzisionstechnik, Gauting, Germany) using magnetic SAM mounting plates and Snow White Plaster No. 2 (Kerr, Salerno, Italy). Mounting the casts in ICP defined this maximum intercuspation as the zero position of the CPI coordinate system. We verified all mounting procedures with split-cast tests, *i.e.* checking the exact fit of the mounting plates after removal of the magnet.

The casts were transferred from the articulator into an electronic CPI (Kondymeter, Vamed, Graz, Austria). The CPI displayed left and right Cartesian coordinates of the condylar spheres in increments of 0.01 mm for ICP, BM, and NM in 3D with 6 measuring gauges (Fig. 1). Two operators conducted the measurements separately under mutual surveillance. Before each measurement, the operators calibrated all gauges to zero while the casts were exactly positioned in ICP. For measurements of BM and NM, the individual bite registrations were carefully placed into the CPI. The operators also put weights onto the centre of the CPI’s upper part for its stabilization during every measurement (5.5 kg, using iron discs of 3 kg and 1.25 kg). Both operators read the displayed xyz values concurrently, transcribed them into an Excel file (Office 2016, Microsoft Corporation, Redmond, USA), and checked for typing mistakes. Each BM and NM position was measured twice and averaged.

### MRI

We thoroughly instructed all participants prior to the MRI examination of both TMJs. Instructions included (i) diligent maintenance of the ICP and the bite registration occlusions during the scans and (ii) avoidance of body movements. Head movements were limited by restriction pads. After scanning the radiologist screened all MRIs for exclusion of pathological alterations.

The technical specifications of the MRI scanner were Magnetom Skyra (Siemens, Erlangen, Germany), field strength 3 T, 16-channel head-neck coil, scans of 5 min and 30 s for sagittal and coronal slices, proton weighted TSE images (TR 2300 ms, TE 10 ms), flip angle 160°, averages 2, concatenation 1, band width 300 Hz/Px, distance factor 10%, image resolution 0.3 × 0.3 × 2 mm voxels, field of view 17 cm. A transversal localizer scan was used for detection of condyle position, paracoronal slices were aligned to the axis of the condylar head. Sagittal slices were adjusted at right angles to the condylar head axis and parallel to the long axis of the mandibular ramus. We omitted dynamic MRI sequences because of their reduced image resolution and movement artefacts. Three MRI runs recorded ICP, NM, and BM condyle positions. The latter were scanned with intraorally repositioned polyvinyl siloxane and ice-water chilled wax bite registrations, respectively. Different bite registration materials prevented their wrong use for the particular scan.

**Figure 2:**
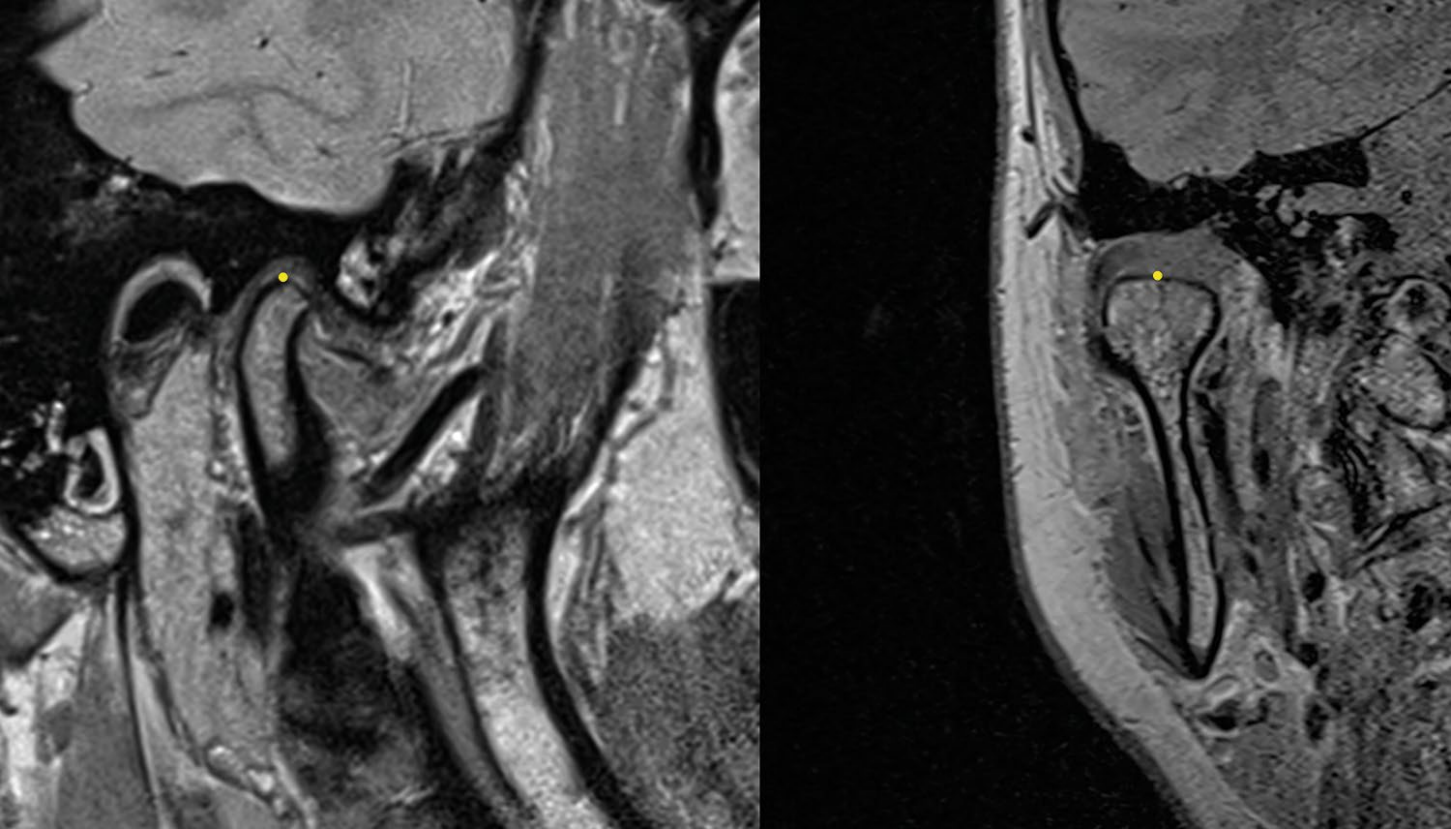
MRI scans in the parasagittal (left) and paracoronal views (right). Yellow dots indicate measuring points on central condyle slices.

3D processing of the MRI data with a medical imaging viewer (Osirix MD; Pixmeo SARL, Geneva, Switzerland) yielded Cartesian coordinates of the measuring points. Zoomed to 600%, we determined the most superior condylar points on central slices of the mandibular condyles in ICP, BM, and NM (Fig. 2). To avoid observer variation, two investigators jointly determined every measuring point twice and transferred the data under mutual control into an MS Excel file.

The same algebraic signs were used for both MRI and CPI coordinate systems. Positive signs indicated cranial location along the superior–inferior axis, lateral location to the right, and posterior position in anterior–posterior direction.

For assessment of measuring point identification errors, the same operators synchronously reiterated measuring point location on every MRI scan of the ICP after 6 days. CPI measurements were repeated following the same protocol and both methods evaluated by intraclass correlation coefficients (ICCs).

### Statistics

All statistical computations were done using R software, version 4.1.1 (R Core Team 2021, Vienna, Austria). Calculations of Euclidean distances have been the square root of the sum of the squared differences in all three dimensions. Prediction ellipses based on Hotelling’s T^2^ were added to 2D scatterplots^13^. Plots of the agreement between CPI and MRI condylar positions are shown with reference to the line of identity and according to Bland and Altman^14^. ICCs and their 95% confidence intervals (CIs) were calculated for intra-rater reliability according to a mixed model formulation^15,16^. The CIs refer to 9999 bootstrap replications of the response residuals, i.e. a semi-parametric bootstrap, and p-values were calculated by inversion of these CIs^17^. The percentage of deviation from the line of identity was calculated in increments of 0.5 mm.

## Results

### Reproducibility of CPI and MRI measurements

ICCs of repeated CPI measurements of all left and right condylar sphere positions showed 0.98 for NM (95% CI 0.96, 0.99) and 0.97 for BM (95% CI 0.93, 0.99) in lateral–medial direction. In anterior–posterior direction, ICCs were 0.93 (0.87, 0.96) for NM and 0.77 (0.61, 0.87) for BM. In superior–inferior direction, NM ICCs equalled 0.87 (0.77, 0.93) and BM 0.80 (0.67, 0.89).

ICCs of repeated MRI measurements showed 0.92 for NM (95% CI 0.85, 0.96) and 0.90 for BM (95% CI 0.82, 0.94) in lateral–medial direction. In anterior–posterior direction, ICCs were 0.95 (0.90, 0.97) for NM and 0.95 (0.92, 0.98) for BM. In superior–inferior direction, NM ICCs equalled 0.92 (0.86, 0.96) and BM 0.94 (0.89, 0.97).

### CPI and MRI condylar positions

**Figure 3:**
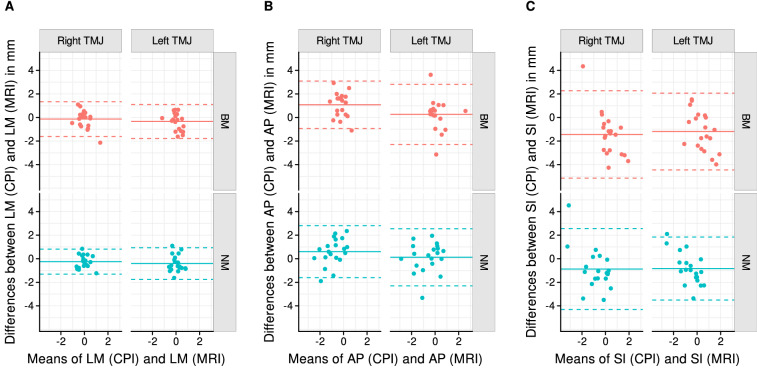
Bland–Altman plots. Illustrations display differences between CPI and MRI condyle position data in millimeters (mm) for lateral–medial (LM), anterior–posterior (AP), and superior–inferior (SI) directions, separately for left and right temporomandibular joints (TMJ) as well as BM and NM positions.

Table 1 shows the descriptive statistics for the left and right 1D condylar positions along the anterior–posterior axis (x), superior–inferior axis (y), and lateral–medial axis (left–right, z) in millimeters, separately for CPI and MRI. The smallest means and medians were found in transverse direction (medial–lateral condylar movement). For all parameters, means and medians were similar whereas they differed more than 0.4 mm between CPI and MRI in superior–inferior direction in all instances. The Bland–Altman plots revealed wide limits of agreement for each spatial direction, ranging from 2.5 mm transversely to 3.5 mm anterior-posteriorly to 5–7.5 mm superior–inferiorly (Fig. 3).

**Table 1:** Descriptive statistics for left and right x, y, and z coordinates of bimanually manipulated (BM) and neuromuscular (NM) condyle positions in millimeters for condylar position indicator (CPI) and magnetic resonance imaging (MRI), n = 20.

	Side	CP	Method	Mean	SD	Min	Q_1_	Median	Q_3_	Max
SI	Left	BM	CPI	− 0.18	0.77	− 2.21	− 0.6	− 0.02	0.29	0.95
			MRI	1.02	1.47	− 1.22	− 0.04	1.17	2	3.62
		NM	CPI	− 1.08	0.65	− 2.18	− 1.54	− 0.97	− 0.77	0.3
			MRI	− 0.25	1.47	− 3.61	− 0.97	0.01	0.81	1.72
	Right	BM	CPI	− 0.41	0.67	− 1.86	− 0.83	− 0.23	0.03	0.88
			MRI	1.03	1.66	− 4.09	0.37	1.06	1.74	3.77
		NM	CPI	− 1.25	0.97	− 3.59	− 1.92	− 0.92	− 0.46	0.1
			MRI	− 0.37	1.71	− 5.38	− 1.16	− 0.06	0.78	1.71
AP	Left	BM	CPI	0.27	0.86	− 1.48	− 0.1	0.05	0.41	2.86
			MRI	0.01	1.03	− 2.19	− 0.57	− 0.28	0.49	2.3
		NM	CPI	− 0.58	1.26	− 2.89	− 1.53	− 0.36	0.57	1.03
			MRI	− 0.71	1.12	− 2.88	− 1.25	− 0.51	− 0.04	1.22
	Right	BM	CPI	0.31	0.61	− 1	0.04	0.39	0.61	1.72
			MRI	− 0.77	0.78	− 2.3	− 1.19	− 0.8	− 0.44	1.24
		NM	CPI	− 0.56	1.17	− 2.88	− 1.5	− 0.46	0.37	1.45
			MRI	− 1.17	0.73	− 2.51	− 1.64	− 1.19	− 0.59	− 0.1
LM	Left	BM	CPI	− 0.11	0.42	− 1.21	− 0.27	0	0.14	0.51
			MRI	0.22	0.66	− 1.15	− 0.17	0.04	0.8	1.33
		NM	CPI	− 0.16	0.52	− 1.04	− 0.44	− 0.09	0.12	0.85
			MRI	0.25	0.55	− 0.87	− 0.1	0.05	0.66	1.25
	Right	BM	CPI	− 0.09	0.43	− 1.28	− 0.26	0.01	0.16	0.53
			MRI	0.05	0.77	− 1.1	− 0.33	− 0.1	0.49	2.41
		NM	CPI	− 0.15	0.5	− 1.05	− 0.28	− 0.1	0.12	0.82
			MRI	0.1	0.52	− 0.69	− 0.2	0.02	0.3	1.63

*SI* superior–inferior, *AP* anterior–posterior, *LM* lateral–medial, *CP* condyle position.

**Figure 4:**
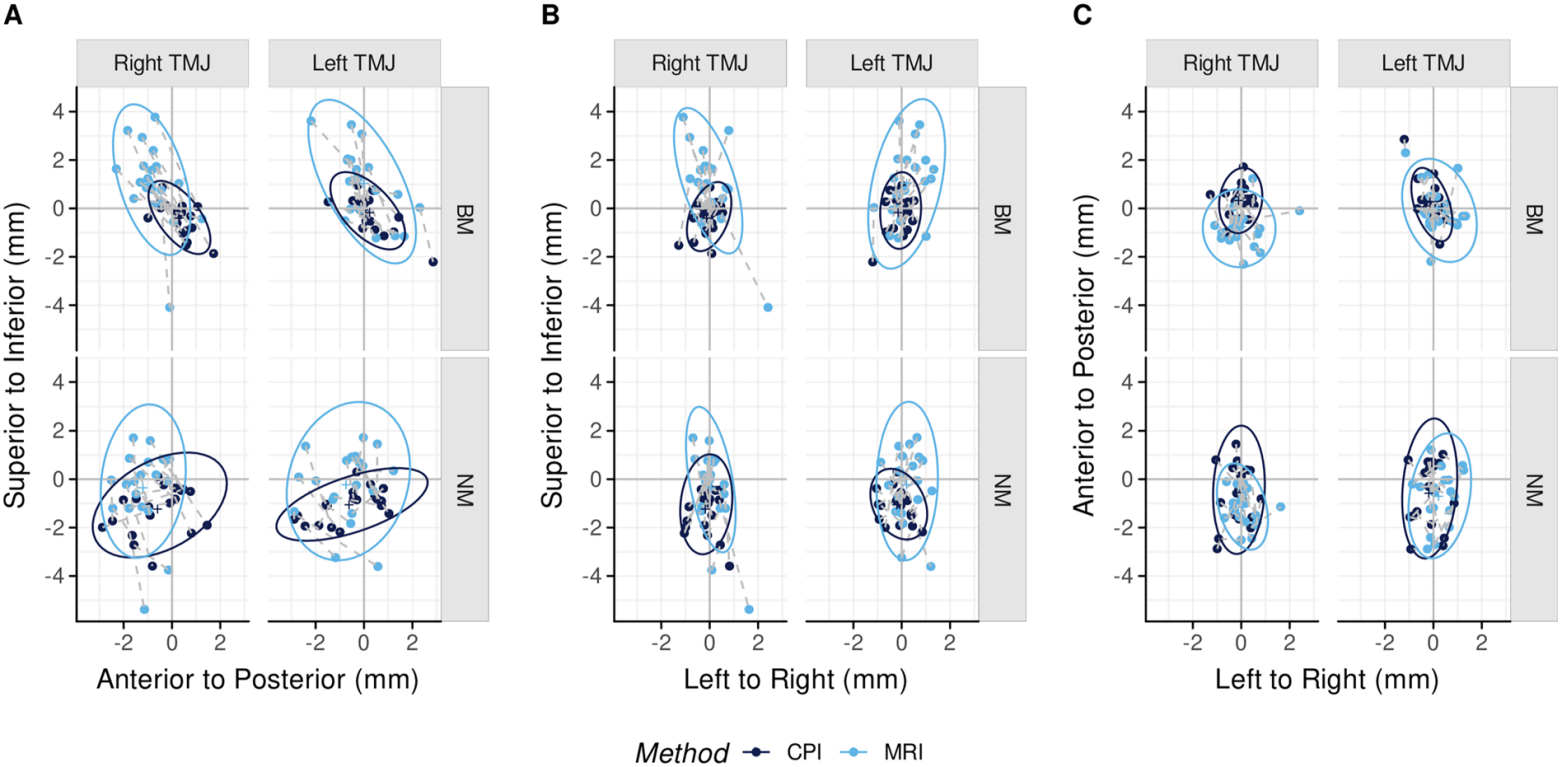
95% data ellipses. CPI data in dark blue, MRI data in light blue. Bimanual manipulation (BM) and neuromuscular position (NM) condylar positions are shown in the sagittal plane (**A**), frontal plane (**B**), and horizontal plane (**C**). Crosses indicate means, dashed lines connect corresponding CPI-MRI condylar positions of individuals.

In 2D, the 95% data ellipses of the sagittal, frontal, and horizontal planes showed little left–right difference. The CPI ellipses were smaller than the MRI ellipses except those of NM in the frontal plane. Ellipses overlapped to varying degrees (Fig. 4).

**Figure 5:**
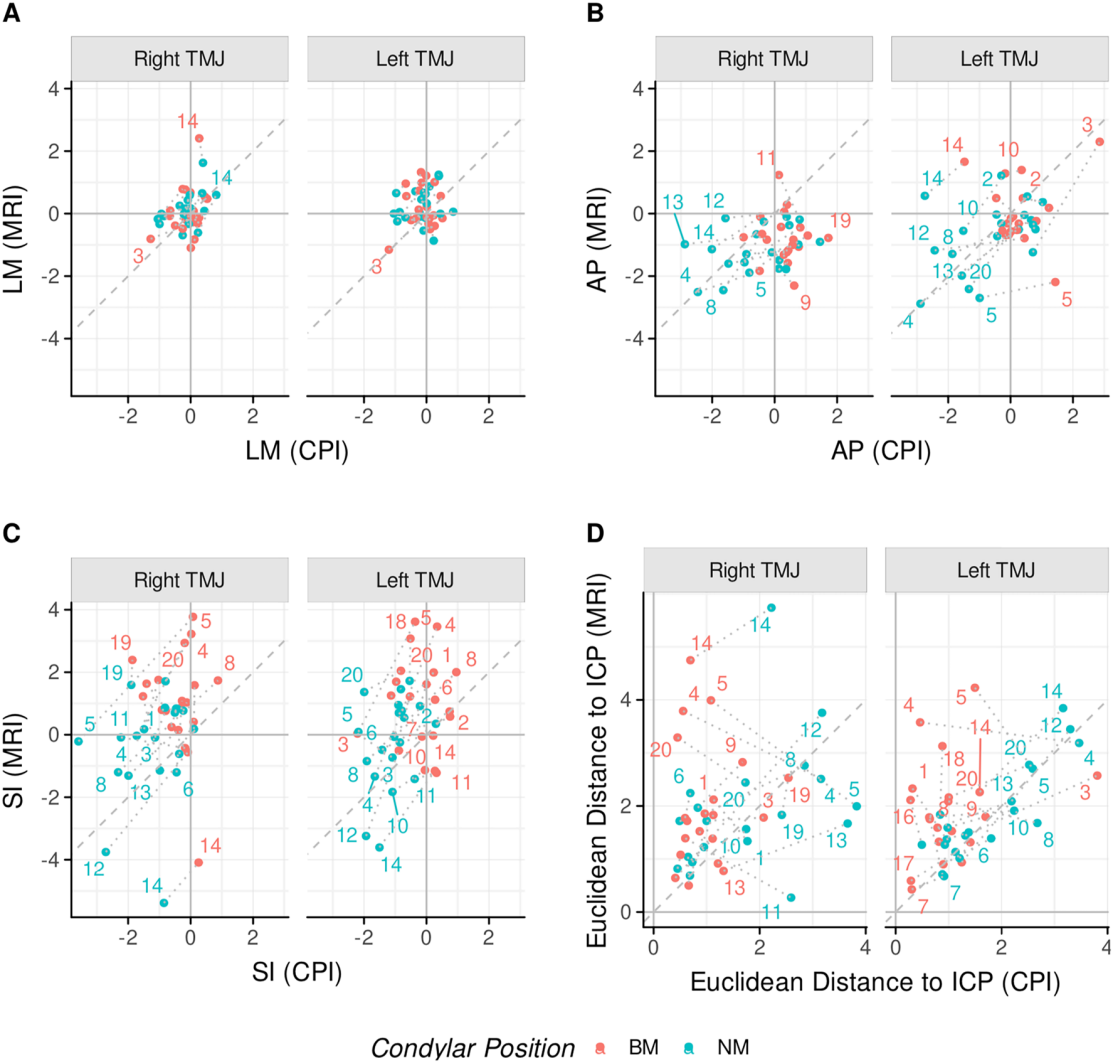
Plot of concordance between CPI and MRI. CPI condylar positions (x-axis) and MRI condyle positions (y-axis), separately for BM (red) and NM (blue) in lateral–medial direction (**A**), anterior–posterior direction (**B**), and superior–inferior direction plane (**C**). (**D**) Illustrates 3D Euclidean distances from intercuspal position (ICP) to BM or NM. Full agreement between CPI and MRI is represented by the line of identity (grey dashed line).

Figure 5 shows the individual methodological concordance of CPI and MRI condyle positions with subject labelled identification numbers. In lateral–medial direction, the BM and NM condylar positions lie close to the line of identity. More scatter exists in anterior–posterior and superior–inferior directions. In terms of Euclidean distances from ICP to BM and ICP to NM, the least inter-individual agreement prevails in Fig. 5D with approximately 1/3 of the points in close proximity to the line of identity.

In 3D, the average Euclidean distances from ICP to BM and ICP to NM differed from 0.09 mm to 1.01 mm between CPI and MRI (Table 2). In the CPI, the left and right 3D distances between BM and NM averaged 1.7 ± 1.11 mm (median 1.63) (n = 40). The corresponding MRI distances between BM and NM showed 1.82 ± 1.22 mm (median 1.49).

**Table 2:** Descriptive statistics for left and right 3D Euclidean distances of bimanually manipulated (BM) and neuromuscular (NM) condyle positions in millimeters, separately for CPI and MRI (n = 20). Abbreviation: CP condylar position.

Side	CP	Method	Mean	SD	Min	Q_1_	Median	Q_3_	Max
Left	BM	CPI	1.03	0.79	0.28	0.59	0.89	1.29	3.81
		MRI	1.92	0.96	0.43	1.32	1.79	2.28	4.23
	NM	CPI	1.75	0.94	0.49	0.95	1.35	2.54	3.46
		MRI	1.84	0.9	0.68	1.27	1.55	2.24	3.84
Right	BM	CPI	1.01	0.56	0.41	0.6	0.92	1.15	2.54
		MRI	2.02	1.17	0.5	1.31	1.78	2.6	4.75
	NM	CPI	1.78	1.13	0.45	0.72	1.74	2.66	3.83
		MRI	1.91	1.21	0.27	1.18	1.72	2.29	5.74

### Methodological agreement

When the MRI xyz coordinates of BM were superimposed onto the BM coordinates of the CPI, all distances between the paired BM positions averaged 2.37 ± 1.3 mm (median 2.19, n = 40). Superimposition of MRI and CPI NM positions yielded 2.06 ± 0.93 mm (median 1.9, n = 40).

Observing the 3D distances between corresponding CPI and MRI measuring points, the percentage of agreement within 0.5 mm was 2.5%. Approximately 11% were within 1 mm, 28% within 1.5 mm, and 50% within 2 mm. Table 3 shows the percentages up to 5 mm difference, separately for BM and NM positions.

**Table 3:** Superimposition of CPI and MRI data.

Condyle position	0.5 mm	1 mm	1.5 mm	2 mm	2.5 mm	3 mm	4 mm	5 mm
BM	2.5%	15%	30%	40%	62.5%	67.5%	87.5%	97.5%
NM	2.5%	7.5%	25%	60%	77.5%	87.5%	92.5%	100%

Percentage of measuring points within categories of 0.5 to 5 mm of 3D Euclidean distance between corresponding CPI and MRI points, separately for bimanual guidance (BM) and neuromuscular technique (NM), n = 40.

Using the ICC for methodological comparison, the coefficients ranged from 0.03 to 0.66. Table 4 lists the coefficients, the 95% CIs, and the derived p-values, which signified statistical difference between both methods (p = 0.0012 and p < 0.001).

**Table 4:** Interclass correlation coefficients (ICCs) of Euclidean 3D distances and 1D data for comparison between MRI and CPI.

	ICC	CI_0.025_	CI_0.975_	p
Eucl ICP-NM	0.66	0.42	0.8	0.0012
Eucl ICP-BM	0.57	0.32	0.74	< 0.001
AP (NM)	0.48	0.22	0.68	< 0.001
AP (BM)	0.03	0	0.17	< 0.001
LM (NM)	0.4	0.15	0.64	< 0.001
LM (BM)	0.36	0.11	0.58	< 0.001
SI (NM)	0.35	0.1	0.6	< 0.001
SI (BM)	0.13	0	0.38	< 0.001

P-values refer to the hypothesis ICC = 1, n = 40.

*CI* confidence interval, *Eucl* Euclidean distance, *ICP* intercuspal position, *NM* neuromuscular position, *BM* bimanual manipulation, *AP* anterior–posterior, *LM* latera–medial, *SI* superior–inferior.

## Discussion

Indirect deductive methods are used when invasive observation is not clinically feasible. Regarding the TMJ, articulators and CPIs simulate occlusal relationships and extrapolate their effects on mandibular condyle position. The degree of truly depicting anatomical conditions with a CPI can be evaluated with established but complex imaging techniques such as MRI or CT. In our study, the ICCs of the MRI and CPI measurements of condylar position showed sufficient reproducibility for each particular method. In the Bland–Altman plots, the values scattered above and below zero, indicating no existing bias of one method versus the other. However, the limits of agreement were wide in the plots, the results therefore ambiguous and without trend but quite consistent variability. Due to the interclass correlation coefficients and their statistical significance, our assumption of no difference between CPI and MRI results had to be rejected. The percentage of CPI-MRI agreement within 0.5 mm was 2.5% only, namely for both BM and NM positions. Greater spatial differences between corresponding MRI and CPI measuring points were almost linear and parallel in their incremental increase of the percentages up to 5 mm, regardless of manual guidance or unguided technique.

Explanations for different outcomes between CPI and MRI include specific characteristics of biological entity and mechanical device. Biological features refer to joint tissue resilience, individual condyle and fossa morphology, neuromuscular action, and combinations of condylar rotation with translation even in initial opening or final closing^18–20^. The medial bony border of the glenoid fossa may limit medially directed condylar movements and cause vertical excursions instead (Fig. 4B). These reasons may also explain that most CPI data ellipses were smaller than the MRI ellipses, particularly in the frontal and sagittal aspects. In other words, the lesser the joint play, the higher was the concordance between MRI and CPI.

The CPI condylar ball has a substantially different shape than the condylar head. Consequently, the measuring points of both objects are diverse. Another issue relates to the anatomical facebow transfer, which offers an approximation of the spatial relationship between maxillary dentition and TMJ hinge axis. Errors from using arbitrary hinge axes instead of kinematic axes and individual variation in intercondylar distance may have contributed to different readings between CPI and MRI^1^. Finally, recumbent *vs.* supine body postures may have affected condyle position during data collection^21^.

Searching for previous comparisons of CPI and MRI data, we found a single study on articulator mounting and MRI dating from 1993^22^. This study investigated leaf-gauge centric relation and maximum intercuspation with both methods. Although the applied methodology differed from our study in detail, the correlation coefficients specified “*a weak relationship between MRI and the articulator*”, too. These correlation coefficients ranged from 0.04 to 0.55^22^ and do not contradict the results of the present study.

As the CPI mostly scaled down the 95% data ellipses in our study, clinicians must expect more positional differences in vivo than in vitro. This shortcoming indicates restrictions of mechanical instruments in replicating functions of biologic systems^23^. Moreover, the ideal condyle position “*has never been found*”^24^ and condyle positions of asymptomatic normals were reported to be concentric in about 50% but posterior or anterior in about 25% each^18,22^. Thus, a single conceptual tenet of where the condyle should be located when in occlusion is questionable. Without using adequate imaging techniques, condyle position is unknown in habitual occlusion and unpredictable in diagnostic reference positions^7^.

MRI applications are not practical in daily routine. Without claim for overall scientific validity, the CPI may be a useful instrument for practitioners under consideration of existing errors. Accepting CPI-MRI agreement within 2 mm as a compromise^25–27^, the CPI will cover approximately 50% of the MRI condylar positions but still neglect the other half.

This uncertainty about the real condylar position does not dismiss the responsibility to strive for precise treatment of occlusion. Dimensions of the dentition should match the therapeutic optimum within individual tolerance. Favorable condyle positions induce minimal biomechanical stress without degenerative alteration^24^. The need for higher or lower degrees of truly replicated anatomy will also depend on the very clinical situation. Less precision might be acceptable for acrylic resin splints than for orthognathic surgery or entire occlusal restorations. Altogether, if CPI values are used, caution must prevail in the interpretation of condylar position.

Limitations of our study address a selection bias by enrolling dental students only. The impracticality of repeating the MRI examination in the same individual implies absence of overall MRI reproducibility data. Repeated measurements of scans from a single MRI run may have overestimated observer reliability and may have contributed to the discrepancy between MRI and CPI measurements. Our sample size was too small for detection of gender differences and left–right asymmetries. In spite of careful split-cast testing, minor mounting errors may have affected the CPI readings.

## Conclusions

Using data from asymptomatic young adults, mandibular condyle positions differed significantly between the in vivo condition depicted by MRI and the in vitro extrapolation from articulator condylar sphere movements (CPI). Methodological agreement within 0.5 mm occurred in 2.5% only. The present study pinpoints unpredictability and huge variation of condylar positions. Exact knowledge of the actual condylar position requires imaging.

## Data Availability

The datasets generated and analyzed during the current study are not publicly available for the time but available from the corresponding author on reasonable request.

## References

[1] Wood DP, Korne PH (1992). Estimated and true hinge axis: A comparison of condylar displacements. Angle Orthod..

[2] Graber, T. M. & Vanarsdall, R. L. *Orthodontics. Current Principles and Techniques.* 211 (Mosby, 1994).

[3] Crawford SD (1999). Condylar axis position, as determined by the occlusion and measured by the CPI instrument, and signs and symptoms of temporomandibular dysfunction. Angle Orthod..

[4] Smith V, Williams B, Stapleford R (1992). Rigid internal fixation and the effects on the temporomandibular joint and masticatory system: A prospective study. Am. J. Orthod. Dentofac. Orthop..

[5] Keshvad A, Winstanley RB (2001). An appraisal of the literature on centric relation. Part III. J. Oral Rehabil..

[6] Bag AK (2014). Imaging of the temporomandibular joint: An update. World J. Radiol..

[7] Kandasamy S, Boeddinghaus R, Kruger E (2013). Condylar position assessed by magnetic resonance imaging after various bite position registrations. Am. J. Orthod. Dentofacial Orthop..

[8] Dawson PE (1973). Temporomandibular joint pain-dysfunction problems can be solved. J. Prosthet. Dent..

[9] Hobo S, Iwata T (1985). Reproducibility of mandibular centricity in three dimensions. J. Prosthet. Dent..

[10] Brill N, Tryde G (1974). Physiology of mandibular positions. Front. Oral Physiol..

[11] Tripodakis AP, Smulow JB, Mehta NR, Clark RE (1995). Clinical study of location and reproducibility of three mandibular positions in relation to body posture and muscle function. J. Prosthet. Dent..

[12] Bumann A, Lotzmann U (2002). TMJ Disorders and Orofacial Pain..

[13] Fox J (2002). An R and S-Plus Companion to Applied Regression.

[14] Bland JM, Altman DG (1986). Statistical methods for assessing agreement between two methods of clinical measurement. Lancet.

[15] Rousson V, Gasser T, Seifert B (2002). Assessing intrarater, interrater and test-retest reliability of continuous measurements. Stat. Med..

[16] Bates D, Mächler M, Bolker B, Walker S (2015). Fitting linear mixed-effects models using lme4. J. Stat. Soft..

[17] Davison AC, Hinkley DV (1997). Bootstrap Methods and Their Application.

[18] Pullinger A (2013). Establishing better biological models to understand occlusion. I: TM joint anatomic relationships. J. Oral Rehabil..

[19] Lundeen HC (1974). Centric relation records: The effect of muscle action. J. Prosthet. Dent..

[20] Rinchuse DJ (1995). Counterpoint: A three-dimensional comparison of condylar change between centric relation and centric occlusion using the mandibular position indicator. Am. J. Orthod. Dentofac. Orthop..

[21] Lee CY, Jang ChS, Kim JW, Kim JY, Yang BE (2013). Condylar repositioning using centric relation bite in bimaxillary surgery. Korean J. Orthod..

[22] Alexander SR, Moore RN, DuBois LM (1993). Mandibular condyle position: Comparison of articulator mountings and magnetic resonance imaging. Am. J. Orthod. Dentofac. Orthop..

[23] Tamaki K, Celar AG, Beyrer S, Aoki H (1997). Reproduction of excursive tooth contact in an articulator with computerized axiography data. J. Prosthet. Dent..

[24] Ueki K (2012). A hypothesis on the desired postoperative position of the condyle in orthognathic surgery: A review. Oral Pathol. Oral Radiol..

[25] Alkhayer A, Piffko J, Lippold C, Segatto E (2020). Accuracy of virtual planning in orthognathic surgery: A systematic review. Head Face Med..

[26] Tonin RH (2020). Accuracy of 3D virtual surgical planning for maxillary positioning and orientation in orthognathic surgery. Orthod. Craniofac. Res..

[27] Baan F (2021). Virtual occlusion in orthognathic surgery. Int. J. Oral Maxillofac. Surg..

